# Institutional core facilities: prerequisite for breakthroughs in the life sciences: Core facilities play an increasingly important role in biomedical research by providing scientists access to sophisticated technology and expertise

**DOI:** 10.15252/embr.201642857

**Published:** 2016-07-13

**Authors:** Doris Meder, Mònica Morales, Rainer Pepperkok, Ralph Schlapbach, Andreas Tiran, Geert Van Minnebruggen

**Affiliations:** ^1^ Core for LifeAssociation for the Advancement of Life Sciences by Core Facilities; ^2^ Max‐Planck‐Institute of Molecular Cell Biology and GeneticsDresdenGermany; ^3^ Centre for Genomic RegulationBarcelonaSpain; ^4^ EMBL HeidelbergHeidelbergGermany; ^5^ Functional Genomics Center ZurichETH Zurich/University of ZurichZürichSwitzerland; ^6^ Vienna Biocenter Core Facilities GmbHVienna Biocenter (VBC)ViennaAustria; ^7^ VIBZwijnaardeBelgium

**Keywords:** S&S: Technology, S&S: Careers & Training, S&S: Economics & Business

## Abstract

Core facilities have become an important resource in biomedical research, providing scientists with access to sophisticated instrumentation and expertise. To enable scientists to perform ever more complex and difficult experiments, core facilities not only need to constantly upgrade technology and expertise, but also cooperate and pool their assets.

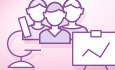

Scientific progress often goes hand in hand with technological advances and interdisciplinary research. Major breakthroughs in the life sciences, such as the deciphering of whole genomes, stem cell therapy or precision medicine, are the result of both new technologies and joint efforts of biologists, physicists, mathematicians and computer scientists. Moreover, each of these accomplishments would not be possible without support infrastructures that provide specific technologies and expertise. Such high‐end research infrastructures, which are often consolidated as core facilities, have helped to foster a collaborative research environment that is crucial for competitive interdisciplinary science and have become an integral part of life science research. The current third biomedical (r)evolution, manifested by the ever increasing speed of technological innovations, means that an individual researcher can no longer afford and master all state‐of‐the‐art techniques. In the current life sciences ecosystem, core facilities are essential and the only means of providing cutting‐edge technologies and expertise in an affordable manner.

Based on our experience operating core facilities, we would like to go even further and take the core facility concept beyond single institutions towards institutional alliances. Providing and maintaining all technologies necessary for the interdisciplinary approaches employed by scientists at leading research institutes have become difficult to impossible at the institutional level. Research projects become more technologically challenging and more expensive. At the same time, technologies turn over ever faster, which imposes a financial burden on the institute and creates a need to find expert scientists to implement, run, improve and adjust those technologies to researchers’ needs. An institute has to focus on a few areas in which it will strive to be at the cutting edge and commit a continuous investment in order to stay there, but it also needs to guarantee access to other technological platforms that cannot be provided in‐house. With this in mind, Core for Life (www.coreforlife.eu) was established in 2012 as a strategic alliance between six institutes that have long‐standing experience in running institutional core facilities to share technologies and to coordinate their investments. Sharing knowledge and expertise, conducting benchmarking studies and developing training curricula are further central activities of the alliance.

The concept for core facilities in the life sciences was boosted by the expensive sequencing technologies that emerged in the early 2000s. After the human genome was sequenced, many research institutions acquired DNA sequencers and hired experts to keep pace with the rapid progress in this field. This started a paradigm shift from science driven by individuals to team‐oriented science where resources and knowledge are pooled. During the post‐genomic era, several leading institutes started full‐fledged core facilities programmes that constituted the institute's centralized research infrastructure. These core facilities are usually open to internal and external users, and accessible on a fee‐for‐service basis, although the pricing policies differ considerably between institutes. This model allows research scientists from any institution access to sophisticated and expensive technologies that would be hard or impossible for their institute to provide for each research group independently.

A core facility is a collaborator who will not say ‘no’, unless there are technical feasibility concerns.

The mission of academic core facilities is to provide expert services and consultation. They act as support units, to which individual scientists outsource technology‐demanding projects that require expertise beyond that of the research laboratory. The type of core facilities we refer to in this article generally conforms to the following principles: it is an independent entity that is not attached to a research group; it is sufficiently large to allow for strategic and flexible deployment of personnel and equipment to offer a range of applications at reasonable turnaround times; scientists from different laboratories, departments or institutes can use the technology; and it is funded via a combination of user fees and institutional funds to support cutting‐edge workflows and novel methods that require investments into implementation and development. The core facility staff will not choose projects or users according to individual or *ad hoc* criteria, but will evaluate each project on the basis of technical feasibility. A colleague once said: “A core facility is a collaborator who will not say ‘no’, unless there are technical feasibility concerns”.

Typically, core facilities have established standard procedures for routine services, such as protein identification from a gel band by mass spectrometry or genotyping of transgenic animals, but quite often they have to develop specific protocols to meet the users' needs. Some services start with a sample and end with sending the data to the user, but many workflows extend to discussing complex experimental questions among facility staff and researchers. Consequently, facility staff is involved as early as the planning stages of the project (which technology should be employed, which controls and how many repetitions are necessary in order to achieve meaningful results, how the sample needs to be acquired and prepared to work with the technology chosen and so on) and as late as analysing the data for the user and even providing the plots for publication. Generally, there is no one‐fits‐all approach and the services are determined by the nature of the technology and the needs of the users.

Core facility operational models range from “user laboratories” to “all‐inclusive services” (Fig 1). User laboratories typically provide access to equipment, and technical experts who advise users which piece of equipment would be best suited for their projects and provide training to use the equipment properly. Light microscopy facilities are typically organized in this way, and the model can be applied to any centralized equipment that requires technical supervision and user training, such as flow cytometry instruments, mass spectrometry and chromatography equipment, pipetting robots, q‐PCR machines and so on. In contrast to user laboratories, the staff at “all‐inclusive” facilities will design and execute the experiments, and/or perform data analysis for the user.

**Figure 1:**
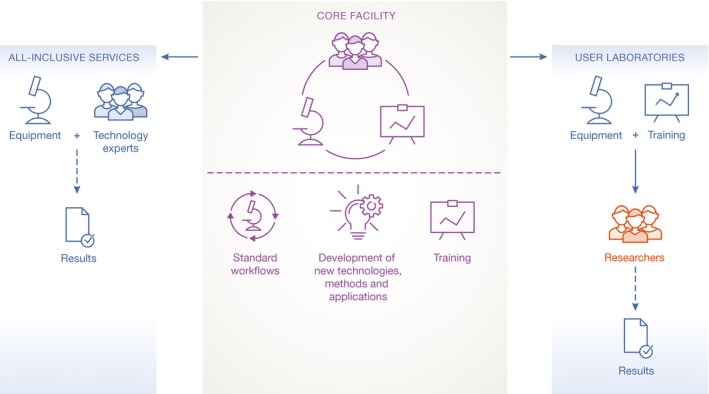
Core facility operational models range from “user laboratories” to “all‐inclusive services” Both comprise equipment and technology experts and provide training, perform standard workflows and in most cases implement or develop new technologies. In the case of “all‐inclusive services”, the researchers provide their sample to the core facility and receive the results. The core facility technology experts perform the experiments on the core facility's equipment and may even analyse the data. In the case of “user laboratories”, the researchers come to use the core facility's equipment on their own after they have been trained and advised by the core facility's technology experts.

Core facility operational models range from “user laboratories” to “all‐inclusive services”.

In addition to such core facilities, other formats of technology platforms exist that are distinct in scale and/or in their operational model. By way of example, the European Bioinformatics Institute (EBI) develops software tools and databases, which it makes available to the research community at large. The Sanger Institute operates genomics platforms that support the institute's own research, as well as collaborative studies and consortia. Another example is the Beijing Genomics Institute, which operates like a contract research organization and provides mass sequencing services, but without individual support for experimental planning and data analysis.

Core facilities enable scientists to design their studies using multiple technologies that they otherwise could not afford or manage on their own. In particular, newly established research groups can kick off their research much faster by accessing state‐of‐the‐art technologies and incorporating core facility staff and equipment into their projects. For recruiting young scientists at the student, postdoc and especially at the young group leader level, access to core facilities is a great asset. However, some group leaders still fear that sharing instruments they crucially depend on with their colleagues will impede their research. A typical example is a research group that uses live imaging and would acquire their own microscope rather than using the central microscopy core facility. But what does this mean in practise? They would have one microscope available to be used by only one student or postdoc at a time. They would have to decide on a certain configuration, which may no longer be the best tool for future projects. And, they would have to live with the same instrument for many years, even if new technologies and better systems become available. Even groups who depend heavily on a certain technology may benefit from shared instrumentation in a core facility where the equipment is properly maintained, and which can be upgraded to more powerful systems as the technology advances.

Core facilities enable scientists to design their studies using multiple technologies that they otherwise could not afford or manage on their own.

There is additional value for both researchers and institutions in sharing technology platforms among research groups. Core facilities are meeting points for scientists from different disciplines and they foster exchange and integration of expertise. A protocol or an instrument that was implemented to serve one project may later serve another user with a different question. Interdisciplinary projects often arise from chance encounters, and core facilities promote such encounters between researchers.

Core facility staff members are also technological mentors for the next generation of scientists. There is a legitimate concern that students and postdocs, who grow up in an environment that provides experts and routine services at their disposal, will be incapable of performing any experiments themselves. It is therefore important for core facility staff to engage in PhD training courses and workshops and offer tutorials at different levels of expertise to make scientists familiar with the technologies. These courses are also entry routes for new core facility staff and therefore critical to sustainability.

While the main mission of core facilities is to support academic research, for‐profit organizations are also increasingly interested in accessing their services. Agro‐, biotech‐ and pharmaceutical companies have been downscaling their research activities and, in turn, started to outsource specific research lines to academic partners. There are a number of reasons for companies to view core facilities in academic institutions as suitable partners: the technology experts provide a sounding board for the company's ideas and can act as consultants; many core facilities meet the expectations of industry better than research groups, as core facility staff is used to working according to standard operating procedures, to adhere to timelines and to budget and to account for the work they perform; and core facilities can help agro‐, biotech‐ and pharmaceutical companies bridge the gap in the early phases between academic and translational research.

Science and technology are continuously evolving, and the big challenge for core facilities is to flexibly adapt to a constantly changing research environment. Core facilities therefore depend on an enabling institutional framework, the corner stones of which are long‐term strategic planning for infrastructure investments and facility personnel and close collaboration with other institutional stakeholders—all of which are greatly enabled by a core facility programme that acts as an umbrella for the individual core facility units.

Most of the technologies offered by core facilities depend on expensive equipment. To ensure that it remains state of the art, the institute needs a sustainable investment plan for acquiring, upgrading and maintaining equipment. The latter is especially important and requires a significant budget for maintenance contracts or repairs, investment into new software tools, data storage, computing capacity and so on. In the case of immature technologies, it may require special funds for subsidizing the first projects in order to implement the technology and leverage its full potential. Technologies that become commodity services may be phased‐out and obtained at equal or cheaper rates from commercial providers, thereby freeing up institutional resources. To anticipate these trends and to react to them in time, it is important to revise the overall strategic plan at regular intervals and to integrate the individual strategies of each core facility and to align them with the institutional vision.

Leveraging the full potential of the equipment relies on the expertise of the staff operating it. Service‐oriented expert scientists, who can understand the users' needs, who strive to push the technology and who take pride in making the users successful, are key to a sustainable core facility. How these people can be attracted and how a facility can find the right balance between retaining their expertise and being responsive to change are important questions. Some institutions rely on permanent contracts to reward dedicated staff, while others offer time‐limited contracts to ensure flexibility by bringing in new expertise through regular turnover. Keeping senior technology experts on permanent contracts ensures that the facility can build on their long‐standing expertise. However, when a technology becomes obsolete, their particular expertise may no longer be needed. Job shadowing and mini‐sabbaticals in other facilities are a way to promote cross‐technology training and flexibility and serve to broaden the staff's technical skills. High turnover on the other hand requires the constant rebuilding of teams with changing composition and leadership styles. It is also important that core facility leaders are independent members of the research faculty, that they are hired via a transparent process involving the research faculty and that their performance is regularly evaluated. Even though their role in the institute is different from a research group leader, providing them with group leader status or technical professorships would appreciate their role and is often a prerequisite for raising extramural funds for technology development and implementation. In the end, building and maintaining staff through ongoing training is as important as maintaining the equipment.

Interdisciplinary projects often arise from chance encounters, and core facilities promote such encounters between researchers.

Also central to the success of a core facility programme is the integration with other institutional stakeholders at the scientific, technological and administrative levels. Scientific and technological exchange between researchers and facility staff can be promoted by joint seminar sessions or by core facility leaders attending group leader meetings. Researchers should be encouraged to bring new developments that they pick up at conferences to the attention of the core facility leader and to bring in their scientific expertise when it comes to evaluating new applications. Some institutes have established technology scouting committees, which continuously spot emerging platforms that could be relevant to the institute's research programme.

Professional support from and productive collaboration with the institute's administration can empower core facilities. A grant office that not only looks out for new project calls, but is also on top of funding opportunities for infrastructure and training, is a great asset. Similarly, a communication department that helps define a marketing plan and advertises the core facilities services is essential to attracting customers and increasing the visibility. A technology transfer office that helps with negotiating for beta‐testing agreements or co‐development with technology providers, and a finance department that is experienced in core facility budgeting, cost calculation and recharge practices, as well as the legal framework related to recharging to grants are key elements to any core facility operation.

Core facilities are the scientists' partners in achieving their research goals. Establishing and maintaining a good dialogue with researchers is a prerequisite to fulfilling the mission of providing researchers with the technologies they need. Surveys, user group meetings, steering and advisory committees, as well as training offers, can be effective tools for collecting user feedback. Surveys can collect feedback from all users and thus provide a general overview. Additional personal interviews allow for collecting in‐depth information on selected topics. In either case, it is crucial to report results to the users, so that they know that their time was well invested.

In addition to surveys, many institutes establish steering or user advisory committees. In most cases, the committee has an advisory function to provide a forum for users interested in a technology to bring in their ideas how to best serve science, and which interesting developments and future requirements they see on the horizon. For the core facility leader, it provides a sounding board that gives feedback on the facility's activities and plans. As a special form of an advisory committee, evaluation boards provide an important source for unbiased, non‐local views on the platform. Owing to the periodic and non‐constant nature of reviews and evaluations, the respective feedback is more strategic by nature, but nonetheless helpful for integrating a broader perspective.

Another valuable strategy for engaging with users is through training. Courses, technology seminars or minisymposia in different technology areas, during which leading international experts present their latest research findings, are a means to reach out to a large user community. Equally important is engaging with local users on an individual basis. Offering technical mini‐sabbaticals for students, postdocs or technicians creates training opportunities in the core facilities, which enables researchers to expand their expertise. Core facilities should consider adding a user laboratory mode to facilitate complex projects that go beyond what the facility can provide. This has a long tradition in microscopy facilities and is now being extended to other technologies such as fluorescence‐activated cell sorting, mass spectrometry or robotics. The crosstalk between core facility staff and users creates a natural feeling of ambassadorship and establishes an environment of trust, in which users and facility staff share knowledge. Scenarios like these are vital to pushing the technologies forward in order to create competitive advantages for researchers.

Interestingly, the main role of core facilities is often perceived as providing access to expensive equipment. Looking at the rapid turnover of technologies and the rate at which new equipment with higher sensitivity, broader dynamic range or new detection principles appears on the market, it is obvious that exclusively relying on the newest piece of equipment cannot be a viable strategy to stay cutting edge. Most institutes will not be able to cope with the scale of financial investment necessary to support a continuous renewal of its full equipment park. Core facilities thus have to join forces and create competitive advantages by other means (Fig 2). These can include leveraging the full potential of their expertise and that of the scientific faculty to create unique applications; combining and adapting already existing technology modules into new workflows; and bridging to non‐biological disciplines.

Core facilities are scientists' partners in achieving their research goals.

**Figure 2:**
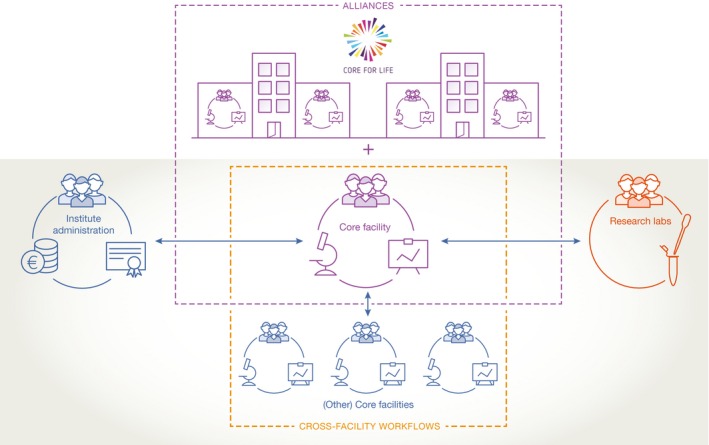
To fulfil their mission of enabling cutting‐edge research, core facilities have to join forces with other core facilities, research groups, institute administration, as well as building alliances across institutes Teamwork between core facilities is required to combine technologies and applications into new cross‐facility workflows. Technology development efforts are usually most effective when a research group and a core facility engage in a joint project. Building alliances across institutes is extremely valuable for exchanging knowledge and expertise and working on common strategies for training, technology scouting, etc.

Creating unique applications requires proactive leadership that looks beyond provision of routine protocols, that is able to identify the hot technologies of the future, which could be relevant to the institute's research mission, and that acquires the necessary expertise—through training of staff and/or collaborations—to test and implement new applications at an early stage. Technology development efforts are usually most effective when a research group and a core facility engage in a joint project and bring in their individual strengths. Examples of such fruitful multi‐party collaborations include a screening workflow for three‐dimensional tissue systems using high‐resolution confocal imaging of organotypic cultures or embryoid bodies, or the use of nanobodies for labelling and measuring protein turnover in correlative light and electron microscopy.

Teamwork between core facilities is similarly required to combine technologies and applications into new cross‐facility workflows. Continuing to build on expertise and experience will create new applications and enable users to answer new questions in emerging scientific disciplines. By way of example, several members of the Core for Life alliance created pipelines for generating transgenic animals using the latest genome engineering technologies which span several facilities: protein expression and genome engineering facilities for producing Cas9, designing the gene editing strategy and developing and testing the expression constructs, the transgenic core or tissue engineering facilities for generating transgenic animals or stem cells and the DNA‐sequencing and bioinformatics facilities for identifying transgenic individuals. Another member's omics facility established collaborative workflows for single‐cell studies together with independent institutional cell sorting facilities. Commercial and self‐built technologies for isolating single cells and expertise for cell classification and manipulation are combined with know‐how at the DNA/RNA and protein analysis levels including bioinformatics and interpretation.

The next step is to bridge across non‐biological disciplines. Life science research is now reaching out to distant fields such as nanotechnology, materials, physics and information sciences. Bringing together scientists and technologists from diverse backgrounds is necessary to foresee the new challenges ahead and to identify ways to tackle them.

Companies are also important partners for developing new applications with benefits for both sides. The core facility and its users receive access to beta‐test instruments while the company receives visibility in a broad user community and direct access to potential customers with a variety of projects that provide feedback for improvements. Examples of successful partnerships are the antibody facility of one of our partner institutions that is set up as a joint venture with an SME (small‐ and medium‐sized enterprise), as well as numerous microscopy and mass spectrometry facilities that partner with instrument providers. Fruitful collaborations could also be established with spin‐offs and small biotech companies, who are granted privileged access to the core facility programme during their start‐up phase. It is also possible that companies host proprietary infrastructure in a specific core facility, thereby minimizing the need for upfront investment into a full operation with several full‐time employees. The core facility can contribute expertise for joint method development and either gains patenting rights on the technology, or the opportunity to offer a newly developed application to its users. Other ways of interactions between industry and core facilities involve joint technology and innovation grants, or the direct collaboration of the facility as a consortium partner in diverse EU FP projects, which include many industrial partners.

As mentioned throughout this article, building and maintaining state‐of‐the‐art core facilities are demanding. Managing the needs of all stakeholders comprises challenges on many levels. Finding the balance between service and research and sizing the core facility correctly is key to any operation, as is effective communication of the facilities' portfolios to scientists. Strategies have to be developed and implemented for attracting and keeping highly trained staff members, for backing‐up machine parks, for implementing emerging technologies and for replacing technologies that have become outdated or commodities. Sustainable costing and pricing models need to be established and adequate funding schemes need to be identified. Last but not least, regularly monitoring and evaluating the core facility's performance is a challenge on its own, as performance indicators are not yet well established.

For core facility managers and staff, it is therefore extremely valuable to exchange such knowledge and expertise. Under FP7, the European Commission started to invest into networking research infrastructures in different member countries. However, efforts to establish a European core facility network that spans disciplines—similar to the Association of Biomolecular Resource Facilities (ABRF) in the USA—have only recently begun to take shape with the establishment of Core for Life and the Core Technologies for Life Sciences (CTLS) conferences to discuss general topics in an open forum.

Cross‐institutional core facility alliances provide possibly the only road to empowering scientists to achieve the highest level of progress for both the future of research and society at large.

Core for Life aims to go beyond these discussions and explore how to coordinate and bundle expertise and resources across institutes. Apart from defining best practices via joint benchmarking projects, alliance partners aim to develop models for capacity sharing and coordinating investments, to jointly scout emerging technologies and to establish an open training network. This can be done only after having built close connections in a small, trusted circle. Via a single contact point in one institute, scientists then have access to the breadth of technologies available in a number of top‐class core facilities at different sites.

The foundation for successful research institutions lies in strengthening and refining existing core facilities that enable cutting‐edge life science research. As the range of methodologies, technology innovation cycles and expert staff knowledge begin to strain the capacity of whole institutions, building networks of institutional core facilities will be the next important step. In an era when access to high‐end technology and know‐how is paramount to research success, cross‐institutional core facility alliances provide possibly the only road to empowering scientists to achieve the highest level of progress for both the future of research and society at large.

## Conflict of interest

The authors declare that they have no conflict of interest.

